# Modulation of the Apoptosis Gene Bcl-x Function Through Alternative Splicing

**DOI:** 10.3389/fgene.2019.00804

**Published:** 2019-09-06

**Authors:** Megan Stevens, Sebastian Oltean

**Affiliations:** Institute of Biomedical and Clinical Science, Medical School, College of Medicine and Health, University of Exeter, Exeter, United Kingdom

**Keywords:** alternative splicing, apoptosis, Bcl-x, RNA-binding proteins, isoform

## Abstract

Apoptosis plays a vital role in cell homeostasis during development and disease. Bcl-x, a member of the Bcl-2 family of proteins, is a mitochondrial transmembrane protein that functions to regulate the intrinsic apoptosis pathway. An alternative splicing (AS) event in exon 2 of Bcl-x results in two isoforms of Bcl-x with antagonistic effects on cell survival: Bcl-xL (long isoform), which is anti-apoptotic, and Bcl-xS (short isoform), which is pro-apoptotic. Bcl-xL is the most abundant Bcl-x protein and functions to inhibit apoptosis by a number of different mechanisms including inhibition of Bax. In contrast, Bcl-xS can directly bind to and inhibit the anti-apoptotic Bcl-xL and Bcl-2 proteins, resulting in the release of the pro-apoptotic Bak. There are multiple splice factors and signaling pathways that influence the Bcl-xL/Bcl-xS splicing ratio, including serine/arginine-rich (SR) proteins, heterogeneous nuclear ribonucleoproteins (hnRNPs), transcription factors, and cytokines. Dysregulation of the AS of Bcl-x has been implicated in cancer and diabetes. In cancer, the upregulation of Bcl-xL expression in tumor cells can result in resistance to chemotherapeutic agents. On the other hand, dysregulation of Bcl-x AS to promote Bcl-xS expression has been shown to be detrimental to pancreatic β-cells in diabetes, resulting in β-cell apoptosis. Therefore, manipulation of the splice factor, transcription factor, and signaling pathways that modulate this splicing event is fast emerging as a therapeutic avenue in the treatment of cancer and diabetes.

## Introduction

Programmed cell death, known as apoptosis, plays a role in cell homeostasis in development and disease. The complex mechanism of apoptosis involves distinct regulatory pathways: the death receptor-mediated (extrinsic) pathway and the mitochondrial (intrinsic) pathway. The extrinsic pathway initiates apoptosis through a death ligand binding to a death receptor, such as tumor necrosis factor (TNF)-α binding to TNF receptor 1 (TFNR1). This results in the recruitment of several death domains, leading to activation of the apoptosis proteins caspase-8 and caspase-10. It is the intrinsic pathway that is regulated by the Bcl-2 family of proteins. The intrinsic pathway is activated by internal stimuli such as DNA damage, oxidative stress, or hypoxia, resulting in a loss of mitochondrial outer membrane (MOM) integrity and release of cytochrome c into the cytoplasm, which forms a complex with Apaf-1 and caspase-9 to form the apoptosome. The apoptosome goes on to activate caspase-3, which then activates cytoplasmic endonucleases (CAD/ICAD) and proteases, leading to degradation of chromosomal DNA, chromatin condensation, cytoskeletal reorganization, and cell disintegration (Elmore, 2007).

Bcl proteins can be either pro- or anti-apoptotic depending on the Bcl-2 homology (BH) domains present (Danial, 2007). Bcl-x is a member of the Bcl-2 family of proteins with multiple BH3 domains. It is a transmembrane protein that lies within the mitochondria and regulates mitochondrial outer membrane permeabilization (MOMP) and release of cytochrome c into the cytoplasm in response to different stimuli (Shamas-Din et al., 2013).

**Figure 1:**
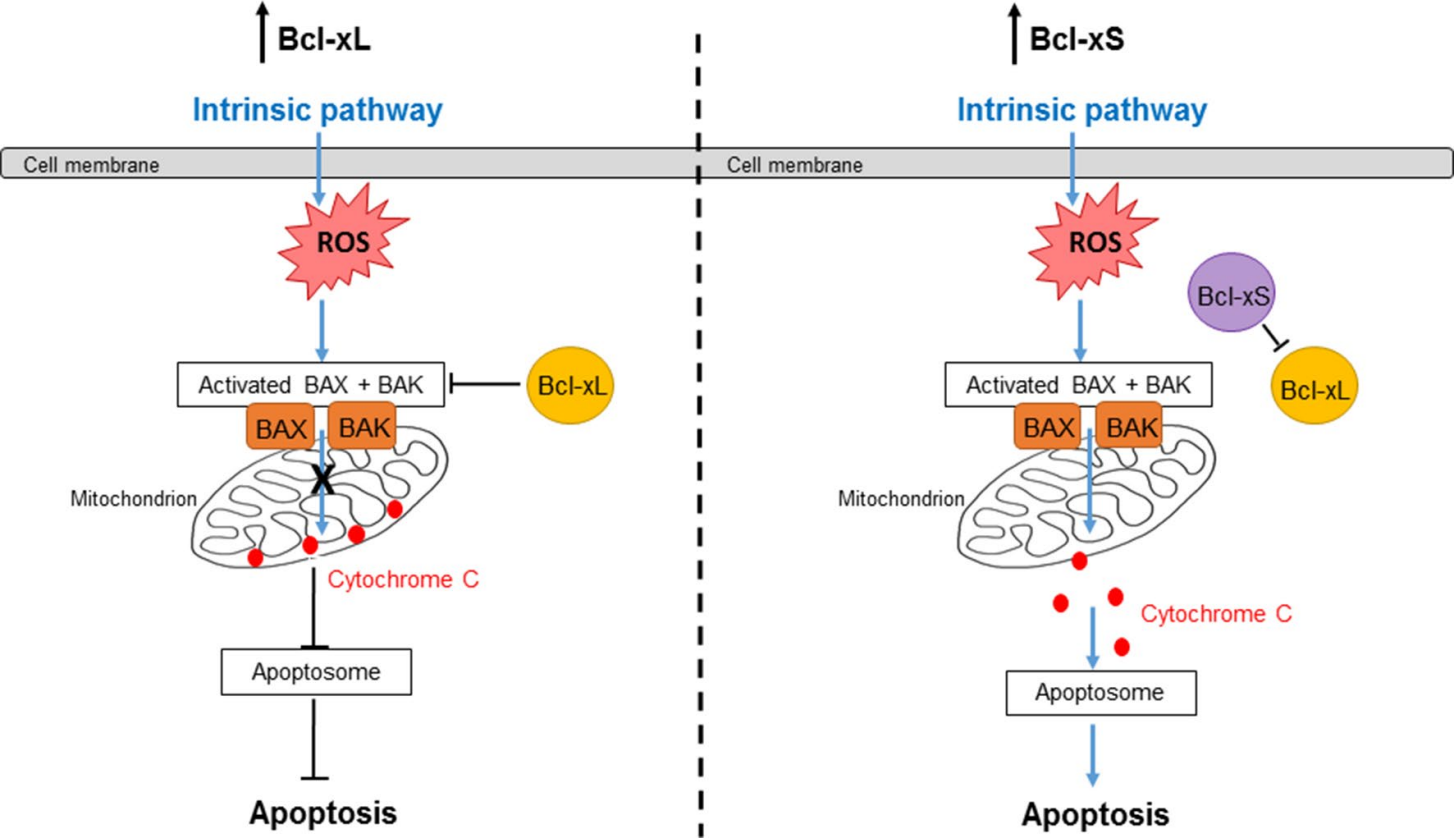
Bcl-xL and Bcl-xS signaling in the intrinsic apoptosis pathway. The intrinsic pathway is activated by internal stimuli such as DNA damage, oxidative stress, or hypoxia. Bcl-xL inhibits the activation of Bax and Bak, preventing a loss of mitochondrial outer membrane (MOM) integrity and release of cytochrome c into the cytoplasm. Therefore, the Bcl-xL isoform is anti-apoptotic. On the other hand, Bcl-xS can inhibit Bcl-xL; thus, the activation of Bax and Bak results in a loss of MOM integrity. Cytochrome c is then released into the cytoplasm, which forms a complex with Apaf-1 and caspase-9 to form the apoptosome. The apoptosome goes on to activate caspase-3, resulting in cell apoptosis. Therefore, the Bcl-xS isoform is pro-apoptotic.

An alternative splicing (AS) event in exon 2 of Bcl-x results in two isoforms of Bcl-x with antagonistic effects on cell survival: Bcl-xL (long isoform), which is anti-apoptotic, and Bcl-xS (short isoform), which is pro-apoptotic (**Figure 1**). This review will focus on how the Bcl-x AS event is regulated in health and disease, as well as discussing how manipulation of Bcl-x splicing could be a potential therapeutic avenue in disease.

## Bcl-x Splice Isoforms

### Mechanism of AS

AS is a key process in genetic diversity through which a single pre-mRNA transcript can give rise to multiple protein isoforms; therefore, AS increases the coding capacity of a gene (Matlin et al., 2005). AS is a highly regulated process; dysregulation can result in cellular dysfunction and disease, including cancer (Oltean and Bates, 2014), diabetes (Stevens and Oltean, 2016; Juan-Mateu et al., 2016), and cardiomyopathy (Guo et al., 2012).

The splicing reaction generally involves the removal of introns from the pre-mRNA and the joining of exons, which is carried out by a macromolecular complex of small nuclear ribonucleoproteins and accessory proteins, known as the spliceosome (Will and Luhrmann, 2011). The spliceosome assembles on splice sites within the pre-mRNA transcript, and the splicing reaction occurs through two transesterification reactions: generation of the branch point and splicing at the 3′ or 5′ splice site (Shi, 2017).

AS is a process where whole exons or parts of exons/introns are included/excluded in the final mRNA transcript. There are four main types of AS event: 1) cassette exon (whole-exon skipping or retention), 2) intron retention (intron remains), 3) alternative 3′ or 5′ splice site (different splice sites within an exon), and 4) mutually exclusive exons (two exons alternate inclusion/exclusion). In some instances, AS can alter the protein that is encoded, which can have an effect on function.

AS is regulated by cis- and trans-acting elements. Cis-acting elements can be divided into four subgroups: exonic and intronic splicing enhancers and exonic and intronic splicing silencers. Cis-acting elements recruit trans-acting splice factors to the splice site to either facilitate or suppress the splicing reaction (Matlin et al., 2005). Splice factors are RNA-binding proteins and include serine/arginine-rich (SR) proteins and heterogeneous nuclear ribonucleoproteins (hnRNPs) (Fu and Ares, 2014). Similar to transcription factors, splice factors are also integral parts of cellular signaling pathways. Modification of splice factor activity and availability through intracellular and extracellular signals results in changes in AS, thus changes in the protein repertoire and cell function.

Transcription factors, termed trans-acting factors, are sequence-specific DNA-binding proteins that bind to response elements. Transcription factors can regulate pre-mRNA splicing through three key mechanisms: 1) influencing transcription elongation rates, 2) binding to pre-mRNA to recruit splice factors, and 3) blocking the association of splice factors with the pre-mRNA (reviewed in Rambout et al., 2018).

### AS of Bcl-x

Bcl-x represents an example of an apoptotic protein whose function is tightly regulated by AS. The Bcl-x gene consists of three exons. Within exon 2, alternative usage of two 5′ splice sites yields two splice variants of Bcl-x, which have antagonistic effects on cell survival. If the proximal 5′ splice site is selected in exon 2, the long isoform (Bcl-xL) is expressed, which has an anti-apoptotic function. On the other hand, if the distal 5′ splice site is selected, the short isoform (Bcl-xS) is expressed, which promotes cell death (Boise et al., 1993) (**Figure 1**).

Regarding the protein, Bcl-xL is a 233-amino acid protein containing four BH domains, a loop between BH3 and BH4, and a transmembrane region. On the other hand, Bcl-xS lacks an internal 63-amino acid segment that contains the conserved BH1 and BH2 domains; therefore, it only contains the BH3 and BH4 domains. The BH1 and BH2 domains are essential for the interaction of Bcl-xL with death agonists, thus suppressing their activity.

Bcl-xL is the most abundant Bcl-x protein and functions to inhibit apoptosis by a number of different mechanisms. It can directly inhibit Bax through binding to it and preventing it from binding to the MOM due to the presence of the BH1 and BH2 domains, induce the translocation of MOM bound Bax to the cytoplasm, and sequester tBid, which is an activator of Bax (Billen et al., 2008; Edlich et al., 2011). As a consequence, Bcl-xL prevents apoptosis through inhibition of MOMP (Shamas-Din et al., 2013). Overexpression of Bcl-xL has been reported to be correlated with increased cell and tissue survival (Yip and Reed, 2008), including pancreatic islet β-cells (Federici et al., 2001; Carrington et al., 2009). In addition, increased expression of Bcl-xL promotes the progression of breast and urothelial cancer (Espana et al., 2004; Yoshimine et al., 2013) and plays a role in chemotherapy resistance (Amundson et al., 2000).

Regulation of the 5′ splice site selected in Bcl-x exon 2 is a critical factor in determining whether a cell is susceptible or resistant to apoptosis. Bcl-xS is the pro-apoptotic isoform of Bcl-x. Bcl-xS can directly bind to and inhibit the anti-apoptotic Bcl-xL and Bcl-2 proteins by forming heterodimers, resulting in the release of the pro-apoptotic Bak (Lindenboim et al., 2001; Plotz et al., 2012). Cell culture studies have shown that increasing the Bcl-xS isoform relative to Bcl-xL can induce apoptosis in cancer cells and pancreatic β-cells (Mercatante et al., 2001; Barbour et al., 2015).

## Regulation of Bcl-x Splice Site Selection

**Figure 2:**
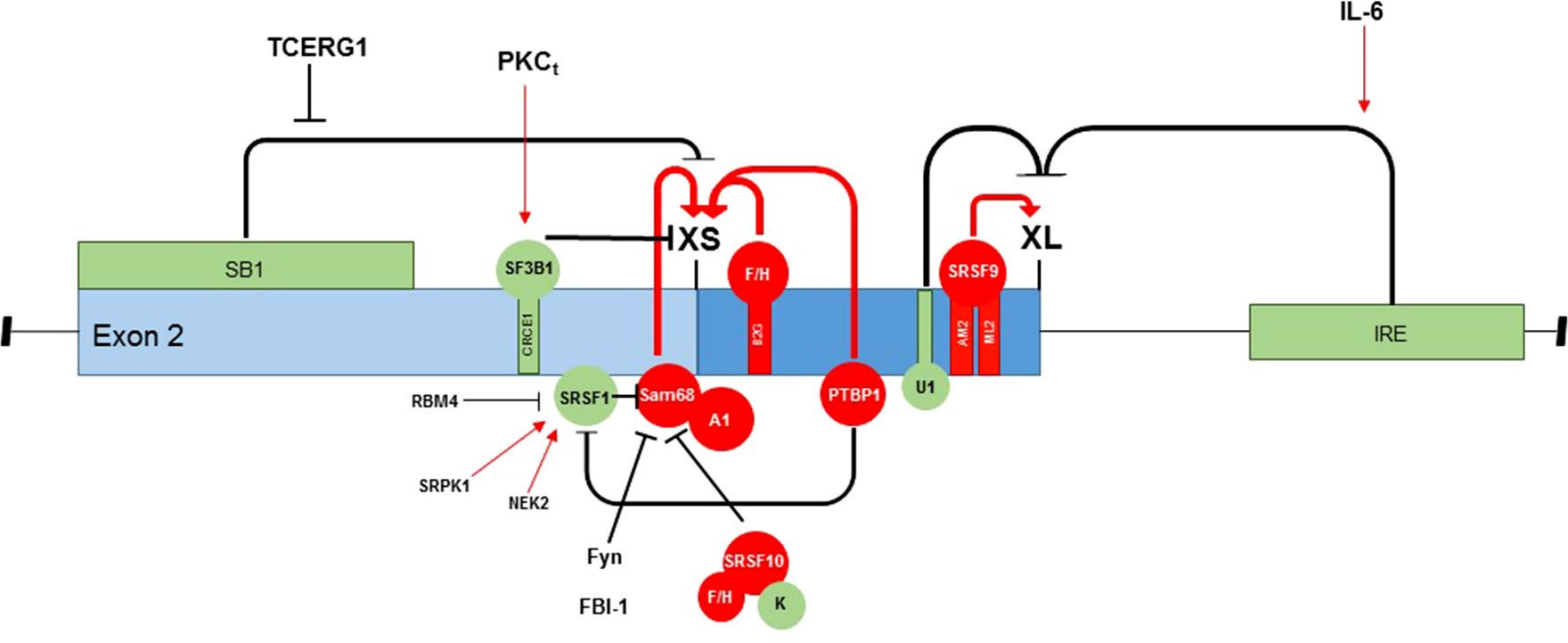
Regulation of Bcl-x exon 2 splice site selection. There are multiple splice factors and signaling pathways that influence 5′ splice site selection in exon 2 of the Bcl-x pre-mRNA, which function to alter the Bcl-xL/Bcl-xS splicing ratio. This schematic summarizes the RNA-binding proteins that can both promote splice site selection (red) and inhibit splice site selection (green), pre-mRNA-binding motifs within the exon, and known signaling pathways involved in splice site selection.

There are multiple splice factors and signaling pathways that influence the Bcl-xL/Bcl-xS splicing ratio (**Figure 2**). SR proteins reported to be implicated in the homeostatic regulation of Bcl-x splicing include SRSF1 (Paronetto et al., 2007; Cloutier et al., 2008), SRSF2 (Merdzhanova et al., 2008), SRSF3 (Bielli et al., 2014a), SRSF7 (Bielli et al., 2014a), SRSF9 (Cloutier et al., 2008), and SRSF10 (Shkreta et al., 2016), as well as the following hnRNPs: A1 (Paronetto et al., 2007), PTBP1 (Bielli et al., 2014a), K (Revil et al., 2009), and F/H (Garneau et al., 2005; Dominguez et al., 2010). RNA-binding proteins include Sam68 (Paronetto et al., 2007), SF3B1 (Massiello et al., 2006), RBM4 (Wang et al., 2014), RBM11 (Pedrotti et al., 2012), RBM25 (Zhou et al., 2008), RBM10 (Inoue et al., 2014), and TRA2β (Bielli et al., 2014a), in addition to the transcription factors TCERG1 and FBI-1 (Montes et al., 2012; Bielli et al., 2014b).

### RNA-Binding Proteins Implicated in Bcl-x AS

The RNA-binding protein Sam68 complexed with hnRNP A1 can bind to the Bcl-x pre-mRNA to promote selection of the distal 5′ splice site and the production of Bcl-xS (Paronetto et al., 2007). This interaction is modulated by the Fyn kinase, which is normally activated through protein kinase C (PKC) signaling (Hsu et al., 2009). Although this collaboration between Sam68 and hnRNP A1 is not thought to make a substantial contribution to Bcl-x splicing under normal growth conditions, where very little Bcl-xS is produced, they have been reported to play a critical role when cells are subjected to DNA damage by treatment with oxaliplatin (Cloutier et al., 2018). Oxaliplatin is proposed to promote tyrosine dephosphorylation on Sam68 by counteracting Fyn kinase activity; therefore, dephosphorylated Sam68 can more readily interact with hnRNP A1 to activate the distal 5′ splice site in exon 2 of Bcl-x (Paronetto et al., 2007). Furthermore, depletion of hnRNP A1 or mutations that impair its interaction with Sam68 attenuated Bcl-xS splice isoform production (Paronetto et al., 2007). In addition to Bcl-x, Sam68 has been also proposed to regulate splice site selection in another apoptosis gene, *BIRC5*, modulating the expression of the anti-apoptotic DEx3 isoform (Gayvan-Cervantes et al., 2017).

Studies have also shown that the interaction of Sam68 with the Bcl-x pre-mRNA is also modulated by SRSF1 and SRSF10. hnRNP A1 is known to compete with SRSF1 to cause switches in 5′ splice sites (Eperon et al., 2000). SRSF1 is suggested to decrease the use of the distal 5′ splice site; in cells treated with SRSF1, the proximal splice site was found to be used exclusively, resulting in the expression of only Bcl-xL (Paronetto et al., 2007). Furthermore, SRSF1 activity is modulated *via* phosphorylation by the kinases NEK2 and SRPK1, both of which have been reported to contribute to apoptosis resistance through the expression of Bcl-xL (Naro et al., 2014). On the other hand, SRSF1 itself is antagonized by the splice factors PTBP1 and RBM4. PTBP1 is reported to bind to a polypyrimidine tract located between the two 5′ splice sites in exon 2; upon binding of PTBP1 to this site, the distal splice site is favored, and Bcl-xS is transcribed (Bielli et al., 2014a). Mechanistically, PTBP1 was reported to displace the binding of SRSF1 to the proximal splice site, therefore repressing the expression of Bcl-xL (Bielli et al., 2014a). RBM4 competes with SRSF1 to bind to the same regulatory element in the Bcl-x pre-mRNA, promoting the expression of Bcl-xS (Wang et al., 2014). SRSF1 is implicated in splice site regulation in many genes, including several other genes in the apoptosis pathway. An example is that of *BIM* and *BIN1*; SRSF1 overexpression has been shown to promote the AS of anti-apoptotic isoforms, thus promoting cell survival (Anczukow et al., 2012).

SRSF10 has been reported to collaborate with hnRNP A1/A2 and Sam68 to drive the DNA damage-induced increase in Bcl-xS (Cloutier et al., 2018). SRSF10 interacts with the repressor hnRNP K and stimulatory hnRNP F/H proteins in normally growing cells, resulting in repression of the Bcl-xS splice site; however, upon DNA damage, SRSF10 becomes dephosphorylated, and its interaction with hnRNP F/H is decreased, thus allowing the stimulatory hnRNP F/H to bind to G-rich regulatory elements located downstream of the Bcl-xS splice site, activating its expression (Shkreta et al., 2016). SRSF2, which is upregulated by the transcription factor E2F1, has also been reported to bind to the same G-rich regulatory elements located downstream of the Bcl-xS splice site, resulting in increased cell apoptosis (Merdzhanova et al., 2008). hnRNP K has been found to bind to CX-rich sequences in a silencer element located upstream of the Bcl-xS 5′ splice site, which results in the repression of Bcl-xS (Revil et al., 2009).

SRSF9 is a splice factor reported to be involved in upregulating the anti-apoptotic splice variant, Bcl-xL. SRSF9 binds to two elements (ML2 and AM2) within the B3 region of the Bcl-x pre-mRNA located immediately upstream of the Bcl-xL donor site, resulting in a shift in splicing to the Bcl-xL 5′ splice site (Michelle et al., 2012). In addition, the B3 region also contains an element that represses Bcl-xL splice site selection, which is bound by U1 snRNP; however, SRSF9 appears to counteract the repressive activity of upstream U1 snRNP-binding sites (Michelle et al., 2012).

In addition to RNA-binding proteins, it has been previously shown that two G-quadruplexes (G4s) form in the Bcl-x pre-mRNA, each of which is close to the two alternative 5′ splice sites (Weldon et al., 2017). Furthermore, G4 ligands have been shown to affect Bcl-x splicing, which act independently at the two splice sites depending on their structure (Weldon et al., 2017).

Components of the exon junction complex (EJC), which is deposited on the mRNA concomitantly with splicing to coordinate mRNA export and surveillance, including eIF4A3, Y14, RNPS1, SAP18, and Acinus, have been reported to regulate Bcl-x splicing, with their knock-down shown to encourage production of a Bcl-xS variant (Michelle et al., 2012). Indeed, Bcl-x was the first mammalian gene for which a role of EJC components in splicing was demonstrated. In addition, depletion of these components of the EJC was also shown to effect the splicing of other apoptosis genes, including *Bim* and *Mc11*, inducing the synthesis of pro-apoptotic splice variants (Michelle et al., 2012).

### Transcription Factors Implicated in Bcl-x AS

TCERG1 is a human nuclear factor implicated in transcriptional elongation and pre-mRNA splicing. TCERG1 has been reported to regulate the splicing of Bcl-x in a promoter-dependent manner; it promotes the splicing of Bcl-xS through the SB1 regulatory element within the first part of exon 2 (Montes et al., 2012). The proposed mechanism for this regulation is that TCERG1 modulates the elongation rate of RNA polymerase II to relieve pausing of the putative polymerase pause site, thus activating the pro-apoptotic Bcl-xS splice site (Montes et al., 2012). In concordance, TCERG1 has been proposed to sensitize cells to apoptosis through changes in mitochondrial membrane permeabilization (Montes et al., 2015). Interestingly, TCERG1 has also been reported to regulate splicing of the apoptosis gene *Fas*/*CD95*, promoting the expression of pro-apoptotic Fas (Montes et al., 2015).

FBI-1 is a BTB/POZ-domain Krüppel-like zinc-finger transcription factor. It has recently been reported to play a direct role in the regulation of AS through its interaction with Sam68, reducing its binding to the Bcl-x pre-mRNA (Bielli et al., 2014b). Like Sam68, FBI-1 is overexpressed in human cancers (Aggarwal et al., 2010; Bielli et al., 2011). Through its interaction with Sam68, FBI-1 promotes splicing of the anti-apoptotic Bcl-xL isoform, thus increasing cell survival (Bielli et al., 2014b).

### Signaling Pathways Implicated in Bcl-x AS

The phosphoinositide 3-kinase (PI3K) pathway has been proposed as a key survival pathway regulating the alternative 5′ splice site selection of the Bcl-x pre-mRNA, increasing Bcl-xL expression in non-small cell lung cancer (NSCLC) cells (Shultz et al., 2012). Protein kinase C_t_ (PKC_t_), atypical PKC, is downstream of PI3K and has been implicated in regulating this AS mechanism and the expression of the splice factor SF3B1, which is an RNA trans-factor that interacts with CREC1 to regulate the 5′ splice site selection of the Bcl-x pre-mRNA (Massiello et al., 2006; Shultz et al., 2012). On the other hand, the classical PKC pathway has been implicated in Bcl-x AS in non-transformed cells (HEK293 cells), where PKC inhibitors were shown to increase the expression of Bcl-xS; such changes in the Bcl-x splicing ratio were not observed when cancer cells were treated with PKC inhibitors (Revil et al., 2009).

Interleukin 6 (IL-6) acts as both a pro-inflammatory cytokine and an anti-inflammatory myokine. Treatment of K562 leukemia cells with IL-6 resulted in a reduction in the Bcl-xL/Bcl-xS ratio; nucleotides 1–176 of the downstream intron were found to be required for the IL-6 effect (Li et al., 2004). It is likely that IL-6 has specific downstream targets that directly regulate Bcl-x splicing; however, the exact mechanism is yet to be elucidated.

Ceramide is an important regulator of cell stress responses and growth mechanisms. A family of ceramide-regulated enzymes known as ceramide-activated protein phosphatases includes the serine/threonine-specific protein phosphatase PP1; endogenous ceramide has been reported to modulate the activity of SR proteins in a PP1-dependent manner (Chalfant et al., 2001). Regarding Bcl-x AS, ceramide has been shown to modulate 5′ splice site selection, increasing the mRNA expression of Bcl-xS, which correlated to an increased sensitization to chemotherapy (Chalfant et al., 2002). More recently, two ceramide-responsive cis-elements within exon 2 of the Bcl-x pre-mRNA have been identified that function to regulate 5′ splice site selection in response to ceramide (Massiello et al., 2004).

## Role of Bcl-x Splicing in Disease

### Cancer

Cancer cells often avoid apoptosis through a change in the expression of genes that control apoptosis, including Bcl-x (Fernald and Kurokawa, 2013). Experimentally increased Bcl-xL expression has been observed in several cancer types, and high Bcl-xL expression is correlated with reduced cellular sensitivity to chemotherapeutic agents (Olopade et al., 1997; Takehara et al., 2001; Mercatante et al., 2002). Altered control of the expression of splice factors, resulting in a change in the balance of pro- and anti-apoptotic splice variants, has also been implicated in cancers. For example, SRSF1 has been shown to be increased in breast cancer (Karni et al., 2007); SRSF1 is associated with an increase in the Bcl-xL/Bcl-xS ratio (Paronetto et al., 2007). On the other hand, SRSF2 is reported to be upregulated in lung cancer (Gout et al., 2012), which results in a decrease in the Bcl-xL/Bcl-xS ratio (Merdzhanova et al., 2008). The reasons for this difference are not yet clear. Furthermore, both FBI-1 and Sam68 are overexpressed in human cancers (Aggarwal et al., 2010; Bielli et al., 2011), which results in an upregulation of Bcl-xL and cell survival (Bielli et al., 2014b).

The splice factor hnRNP K represses the expression of Bcl-xS, suggesting an anti-apoptotic mechanism in cancer cells (Revil et al., 2009). Increases in the expression and changes in the cellular distribution of hnRNP K have been demonstrated in many cancer types, indicating it to be a prognostic marker of cancer (Pino et al., 2003; Carpenter et al., 2006; Chen et al., 2008). It has been proposed that hnRNP K interacts with the phosphatase 2A (PP2A) inhibitor SET to promote tumorigenesis through a reduction in Bcl-xS levels (Almeida et al., 2014).

BC200 is a long non-coding RNA (lncRNA) that has been shown to be upregulated in breast cancer. Interestingly, a knockout (KO) of BC200 suppressed tumor cell growth both *in vitro* and *in vivo* through increased expression of the Bcl-xS isoform (Singh et al., 2016). Therefore, BC200 is proposed to play an oncogenic role in breast cancer through binding to the Bcl-x pre-mRNA and recruiting hnRNP A1/B2 (Singh et al., 2016).

The splicing suppressor RBM4 has recently been implicated in tumorigenesis; its expression is significantly decreased in cancer patients, and its level is positively correlated with improved survival (Wang et al., 2014). Mechanistically, RBM4 antagonizes SRSF1 and upregulates the expression of the pro-apoptotic Bcl-xS isoform, thus acting as a tumor suppressor (Wang et al., 2014).

Therefore, in general, an upregulation of the pro-survival Bcl-xL is often observed in cancer cells, which may be, at least in part, due to the dysregulation of certain splice factors involved in Bcl-x pre-mRNA splicing.

### Diabetes

3Although the evidence thus far for the role of Bcl-x AS in diabetes is limited, it is clear that β-cell apoptosis plays a major role in the pathogenesis of diabetes, which correlates with the increased expression of the pro-apoptotic Bcl-xS splice isoform. Further research is needed to elucidate the mechanisms by which Bcl-x AS is regulated in diabetes to determine the spice factors and signaling pathway involved.

## Manipulation of Bcl-x Splicing as a Potential Therapeutic Avenue

Manipulating the expression of the Bcl-x isoform ratio is emerging as a potential therapeutic avenue in certain disease types. This includes some form of cancers where tumor cells are resistant to chemotherapeutic agents due to the increased expression of anti-apoptotic Bcl-xL and diabetes where β-cells undergo apoptosis correlating to a shift in the splicing ratio to promote the pro-apoptotic Bcl-xS isoform relative to Bcl-xL.

In cancer, one of the key causes of chemoresistance is the resistance of cancer cells to apoptosis (Fulda, 2009). The manipulation of splice factor expression has been shown to sensitize cancer cells to therapeutic treatments, acting either as pro-survival factors that diminish drug-induced apoptosis or as pro-apoptotic factors to potentiate the effects of chemotherapeutics. An example is the anti-apoptotic splice factor SRSF1. Downregulation of SRSF1 in cervical cancer cells with the AURKA kinase inhibitor VX-680 altered the splicing of Bcl-x to increase Bcl-xS, sensitizing the cells to VX-680-induced apoptosis (Moore et al., 2010). Furthermore, silencing SRSF1 in cancer cell lines has been shown to facilitate apoptosis induced by gemcitabine (Adesso et al., 2013). Similarly, hnRNP K has also been reported to interfere with the tumor response to chemotherapeutics. In acute myeloid leukemia cells, a reduction in hnRNP K is required in order for NSC606985, a camptothecin analogue, to trigger cell apoptosis (Go et al., 2009).

Splice-switching oligonucleotides (SSOs) are anti-sense oligonucleotides that hybridize to pre-mRNA sequences, blocking the binding of splice factors, thus redirecting the splicing machinery to an alternative pathway and modifying splicing of the gene. In cancer, a commonly reported therapeutic effect of SSOs is of targeting the Bcl-x pre-mRNA to redirect splicing from Bcl-xL to Bcl-xS, resulting in pro-apoptotic and chemosensitizing effects in various cancer cell lines (Mercatante et al., 2002; Bauman et al., 2009; Bauman et al., 2010; Li et al., 2015). However, the effects of the Bcl-x SSOs appear to vary depending on the expression profile of the target cells, which was suggested to be attributed to the endogenous levels of the Bcl-xL variant; tumor cells with higher endogenous levels of Bcl-xL were reported to be more susceptible to the effects of Bcl-x SSOs, which is likely to be due to the SSO being able to produce enough Bcl-xS to promote apoptosis due to the higher transcription levels of Bcl-x (Mercatante et al., 2001; Mercatante et al., 2002).

Certain chemical classes of G4 ligands, including ellipticine and quindoline derivatives, have been reported to have diverse effects on 5′ splice site usage in Bcl-x; for example, the ellipticine GQC-05 antagonizes the Bcl-xL 5′ splice site and activates the Bcl-xS 5′ splice site, thus inducing cell apoptosis (Weldon et al., 2017). Such ligands may have the potential to switch Bcl-x splicing in a therapeutic manner.

Islet transplantation is fast becoming a realistic alternative treatment option for patients with a brittle form of type I diabetes (Ricordi and Strom, 2004). However, retaining islet viability is a problem. One study reported that delivering the Bcl-xL variant to the islets *via* protein transduction resulted in an improvement in islet viability, thus preserving islets for transplantation (Klein et al., 2004). In addition, insulin-like growth factor 1 (IGF-1) was shown to be protective against type I diabetes in non-obese diabetic mice as shown by reduced β-cell apoptosis resulting from increased expression of the anti-apoptotic Bcl-xL and Bcl-2 (Chen et al., 2004).

A major problem that comes with the manipulation of AS events as a potential therapeutic option is how to target the splicing event within a particular cell type or tissue. It is of major concern that although reducing the Bcl-xS/Bcl-xL ratio in diabetes would have therapeutic effects regarding β-cell apoptosis, a decrease in the Bcl-xS/Bcl-xL ratio at a whole-organism level may promote tumor cell survival. One potential avenue that could be used to address this issue is using exosomes to deliver SSOs or splicing modulating drugs to target tissues. Exosomes are naturally occurring nanovesicular structures that are secreted by most cell types and are suggested to be the “next-generation” carrier for gene therapy. Although there are few publications to date, Alvarez-Erviti et al. (2011) have successfully delivered siRNA to the mouse brain *via* targeted exosomes.

## Conclusion

AS of Bcl-x is a tightly regulated event that determines the apoptotic potential of the cell. In general, most cell types predominantly express the anti-apoptotic Bcl-xL isoform. However, a further upregulation of Bcl-xL expression in tumor cells can result in resistance to chemotherapeutic agents. On the other hand, dysregulation of Bcl-x AS to promote Bcl-xS expression has been shown to be detrimental to pancreatic β-cells in diabetes, resulting in β-cell apoptosis. Therefore, manipulation of the splice factor, transcription factor, and signaling pathways that modulate this splicing event is fast emerging as a therapeutic avenue in the treatment of cancer and diabetes; however, further research is required to investigate whether Bcl-x splicing can be modulated in a cell/tissue-specific manner.

## Author Contributions

MS wrote the manuscript. SO helped with revisions and approved the final version.

## Funding

Funding for this study was supported by grants to SO: British Heart Foundation (PG/15/53/31371), Diabetes UK (17/0005668)

## Conflict of Interest Statement

The authors declare that the research was conducted in the absence of any commercial or financial relationships that could be construed as a potential conflict of interest.
